# Altered Grooming Syntax and Amphetamine-Induced Dopamine Release in EAAT3 Overexpressing Mice

**DOI:** 10.3389/fncel.2021.661478

**Published:** 2021-06-21

**Authors:** Angélica P. Escobar, Jonathan Martínez-Pinto, Francisco Silva-Olivares, Ramón Sotomayor-Zárate, Pablo R. Moya

**Affiliations:** ^1^Facultad de Ciencias, Centro Interdisciplinario de Neurociencia de Valparaíso (CINV), Universidad de Valparaíso, Valparaiso, Chile; ^2^Facultad de Ciencias, Instituto de Fisiología, Universidad de Valparaíso, Valparaiso, Chile; ^3^Facultad de Ciencias, Centro de Neurobiología y Fisiopatología Integrativa (CENFI), Universidad de Valparaíso, Valparaiso, Chile

**Keywords:** EAAT3, EAAC1, *SLC1A1*, dopamine receptor, obsessive–compulsive disorder, microdialysis, striatum, amphetamine

## Abstract

The excitatory amino acid transporter EAAT3 plays an important role in the neuronal uptake of glutamate regulating the activation of glutamate receptors. Polymorphisms in the gene-encoding EAAT3 have been associated with obsessive–compulsive disorder (OCD), although the mechanisms underlying this relationship are still unknown. We recently reported that mice with increased EAAT3 expression in forebrain neurons (EAAT3^*glo*^/CMKII) display behavioral and synaptic features relevant to OCD, including increased grooming, higher anxiety-like behavior and altered cortico-striatal synaptic function. The dopamine neurotransmitter system is implicated in ritualistic behaviors. Indeed, dopaminergic neurons express EAAT3, and mice lacking EAAT3 exhibit decreased dopamine release and decreased expression of the dopamine D1 receptor. Moreover, EAAT3 plays a role on the effect of the psychostimulant amphetamine. As such, we sought to determine if the OCD-like behavior in EAAT3^*glo*^/CMKII mice is accompanied by altered nigro-striatal dopaminergic transmission. The aim of this study was to analyze dopamine transmission both in basal conditions and after an acute challenge of amphetamine, using behavioral, neurochemical, molecular, and cellular approaches. We found that in basal conditions, EAAT3^*glo*^/CMKII mice performed more grooming events and that they remained in phase 1 of the grooming chain syntax compared with control littermates. Administration of amphetamine increased the number of grooming events in control mice, while EAAT3^*glo*^/CMKII mice remain unaffected. Interestingly, the grooming syntax of amphetamine-control mice resembled that of EAAT3^*glo*^/CMKII mice in basal conditions. Using *in vivo* microdialysis, we found decreased basal dopamine levels in EAAT3^*glo*^/CMKII compared with control mice. Unexpectedly, we found that after acute amphetamine, EAAT3^*glo*^/CMKII mice had a higher release of dopamine compared with that of control mice, suggesting that EAAT3 overexpression leads to increased dopamine releasability. To determine postsynaptic effect of EAAT3 overexpression over dopamine transmission, we performed Western blot analysis of dopaminergic proteins and found that EAAT3^*glo*^/CMKII mice have higher expression of D2 receptors, suggesting a higher inhibition of the indirect striatal pathway. Together, the data indicate that EAAT3 overexpression impacts on dopamine transmission, making dopamine neurons more sensitive to the effect of amphetamine and leading to a disbalance between the direct and indirect striatal pathways that favors the performance of repetitive behaviors.

## Introduction

Obsessive–compulsive disorder (OCD) is a psychiatric illness characterized by the performance of repetitive behaviors (compulsions) and the presence of intrusive, anxiety-generating thoughts (obsessions) that affects 2–3% worldwide (Grabe et al., 2000; Angst et al., 2004; Bienvenu et al., 2012). A brain circuit largely implicated in OCD is the cortico-striato-thalamo-cortical loop (CSTC), where an imbalance between the efferent pathways of the striatum (either an overactivation of the striatal direct pathway or an inhibition of the indirect pathway) would lead to repetitive behaviors (Saxena and Rauch, 2000; Saxena et al., 2001). Importantly, the activity of the striatal pathways and its dorsolateral and ventral portions are modulated by dopamine (DA) projections from the ventral tegmental area (VTA) and the substantia nigra *pars compacta* (SNc). At the neurochemical level, striatal alterations on DA release as well as on the expression levels of DA receptors could lead to an imbalance in the fine-tuned regulation of the direct and indirect pathways, contributing to the development of compulsive behaviors. Interestingly, altered expression of DA system proteins in the striatum have been reported in neuroimaging studies of OCD (Denys et al., 2004; Hesse et al., 2005; Moresco et al., 2007). In animals, the DA transporter (DAT) knockdown mice shows an increase in DA extracellular level in the striatum and a repetitive grooming behavior (Berridge et al., 2005). On the other hand, the repeated activation of DA type 2 receptors (D2Rs) produces compulsive checking behavior in rats (Szechtman et al., 1998; Escobar et al., 2015) and mice (Asaoka et al., 2019; Sun et al., 2019). These findings reinforce the notion that altered DA neurotransmission may be involved in the development of repetitive behaviors, a cardinal trait in OCD.

Dysfunctions in glutamate system have also been suggested to contribute to the OCD etiology; for detailed reviews, please see Grados et al. (2013), Escobar et al. (2019), and Robbins et al. (2019). Human genetic studies suggest a role for the *SLC1A1* gene encoding the neuronal excitatory amino acid transporter 3 (EAAT3) in OCD (Arnold et al., 2006; Dickel et al., 2006; Wendland et al., 2009; Wu et al., 2013). EAAT3 is expressed postsynaptically in GABA, glutamate, and DA neurons (Coco et al., 1997; Conti et al., 1998; Sidiropoulou et al., 2001; Underhill et al., 2014), buffering glutamate extracellular levels to regulate the activation of AMPA and NMDA receptors (Scimemi et al., 2009; Jarzylo and Man, 2012; Underhill et al., 2014; Delgado-Acevedo et al., 2019).

Mouse models lacking EAAT3 expression have reported dissimilar results in regard to OCD-like behavior. EAAT3 heterozygous mice were found to have no behavioral or neurochemical alterations relevant to OCD (González et al., 2017). In EAAT3 knockout (KO) mice, early studies found no alterations in anxiety or compulsive-like behaviors at 6–8 weeks old but reported a reduction in self-grooming behavior at 10–12 months old (Aoyama et al., 2006), while a recent study found increased grooming in EAAT3 KO mice at an early age, from postnatal day 14 to 35 (Bellini et al., 2018). Using a different EAAT3 KO mouse model, Zike and colleagues reported a reduction in grooming behavior at 2- to 4-month-old mice (Zike et al., 2017). Based on genetic findings of *SLC1A1* gene variants associated with OCD in a large case-control study and that correlated with higher EAAT3 expression in human brain tissue (Wendland et al., 2009), we recently developed a transgenic model with increased EAAT3 expression in principal forebrain neurons (EAAT3^*glo*^/CMKII) (Delgado-Acevedo et al., 2019). EAAT3^*glo*^/CMKII mice display increased anxiety and compulsive behaviors as well as deficits in cortico-striatal synaptic function, demonstrating that EAAT3 overexpression could be related to the pathophysiology of OCD (Delgado-Acevedo et al., 2019).

Interestingly, EAAT3 is known to impact dopamine neurotransmission. For example, EAAT3 KO mice show a reduction in DA type 1 receptor (D1R) expression and reduced basal DA extracellular levels in the striatum (Zike et al., 2017), suggesting that the decreased grooming behavior found in this model could be a consequence of a decreased activation of the D1R-direct striatal pathway (Zike et al., 2017). In addition, striatal DA release induced by amphetamine and a concomitant grooming behavior are reduced in EAAT3 KO compared with control mice (Zike et al., 2017). On the other hand, Bellini et al. reported an increase in striatal D1R expression in EAAT3 KO mice, which is correlated with higher grooming behavior (Bellini et al., 2018). EAAT3 is expressed in DA neurons (Sidiropoulou et al., 2001; Underhill et al., 2014; Li et al., 2017), and it has been suggested as a neuroprotective effect in late adulthood, since a reduction in EAAT3 expression is related to a reduced number of DA neurons (Berman et al., 2011). Furthermore, it has been shown that the pharmacological effect of amphetamine is mediated by EAAT3 because amphetamine induces the internalization of EAAT3 in DA neurons and potentiates synaptic excitatory glutamate currents (Underhill et al., 2014; Li et al., 2017). Thus, the EAAT3 expression level might regulate the activity of dopaminergic neurons (Li et al., 2017), ultimately impacting on DA release. As dopamine neurons express calcium/calmodulin-dependent kinase 2 promoter (CMKII) (Wang et al., 2013) and the fact that EAAT3^*glo*^/CMKII mice exhibit OCD-like behaviors (Delgado-Acevedo et al., 2019), we sought to determine if EAAT3 overexpression impacts on DA system by analyzing neurochemical, molecular, and behavioral parameters in basal conditions as well as by amphetamine challenge.

## Materials and Methods

### Animals

EAAT3^*glo*^/CMKII is a double transgenic mouse line that overexpresses EAAT3 under the control of CMKII using a cre-LoxP system (Delgado-Acevedo et al., 2019). EAAT3^*glo*^ littermate mice lacking CMKII-cre were used as controls. Mice were housed in the animal care facility at the Facultad de Ciencias, Universidad de Valparaiso, under the care of a veterinarian. Mice were housed in groups of two to five per cage with food and water *ad libitum*, in a light–dark cycle of 12 h (lights on at 7:00 a.m.) in a room with controlled temperature and humidity. Behavioral and surgical procedures were done in mice 3–5 months old. In order to reduce the number of animals used, mice were first tested in behavioral procedures and after at least 14 days to allow for amphetamine washout; *in vivo* microdialysis, immunohistochemistry, or molecular assays were performed in the same animals. All procedures were performed according to the NIH Guidelines and approved by the institutional Bioethics Committee of the Universidad de Valparaiso (BEA138-19).

### Drugs

Amphetamine sulfate was donated by Laboratorio Chile S.A. (Santiago, Chile), and it was dissolved in saline solution at a concentration of 1.5 mg/ml. The dose of amphetamine for behavioral and neurochemical experiments was 5.0 mg/kg subcutaneously (s.c.).

### Behavior

All behavioral procedures were performed in a behavioral room between 9:00 and 14:00 h. Mice were handled for 20 min each day for three consecutive days to allow habituation. Before the behavioral test, mice were acclimated to the behavioral room at least for 1 h. All experiments were performed in blind to the animal’s genotype, and the equipment was cleaned with 5% ethanol solution before and after each test.

#### Locomotor Activity

Mice were placed in the center of an open field arena (40 × 40 × 35 cm high), and basal horizontal locomotor activity was recorded for 45 min. Next, a single dose of amphetamine (5 mg/kg, s.c.) was administered, and activity was recorded for an additional 45 min. Locomotor activity parameters were recorded and analyzed using Noldus Ethovision XT (Noldus Information Technology, Leesburg, VA, United States). Also, from the first 5 min of recording, the time and frequency in the center of the arena (20 × 20 cm) were determined to evaluate anxiety-like behavior. EAAT3^*glo*^ *n* = 11 (6M, 5F); EAAT3^*glo*^/CMKII *n* = 8 (5M, 3F).

#### Grooming

Mice were placed in a clear plexiglass cylinder, 20-cm diameter and 30-cm high, and recorded using a video camera placed in front of the cylinder. Grooming was recorded in basal conditions for 10 min. Immediately after, mice received a single dose of amphetamine challenge (5 mg/kg, s.c.) and were returned to its home-cage for 35 min. Then the mice were placed back in the clean cylinder, and grooming behavior was recorded for the next 10 min; this was done to properly compare the drug exposure time to the locomotor activity experiments. Grooming behavior (number of events and time spent in grooming) was analyzed manually. Each grooming event was classified according to the grooming phase reached (Cromwell et al., 1998; Berridge et al., 2005; Kalueff et al., 2007). Briefly, the grooming chain syntax consists of four patterns of grooming movements: phase 1 consists of a series of ellipse-shaped movements around the nose; phase 2 is defined as a series of movements made by one paw that reach the mystacial vibrissae to below the eye, phase 3 is a series of movements made by both paws simultaneously reaching the ears, and phase 4 is a sustained movement of body licking (Berridge et al., 2005; Kalueff et al., 2007). EAAT3^*glo*^ *n* = 7 (4M, 3F); EAAT3^*glo*^/CMKII *n* = 9 (3M, 6F).

### *In vivo* Microdialysis in Anesthetized Mice

Mice were first anesthetized with isofluorane (3% in 0.5 L/min airflow) in an induction chamber, and then placed in a stereotaxic apparatus with a mask to maintain anesthesia for all the experiment (1–2% isofluorane in 0.5 L/min, airflow) using an animal anesthesia system (model 510, RWD Life Science Co. Ltd., Shenzhen, China). Body temperature was maintained at 37°C. Craniotomy was made according to the following stereotaxic in mm: AP: 1.0, ML: 1.5, according to Paxinos Mouse Brain Atlas. A microdialysis probe (length 2 mm, 0.26 mm diameter, 6 kDa, CMA-11, Harvard Apparatus) was lowered to reach 1.8 mm under the dura, covering the dorsal striatum. The microdialysis probe was continuously perfused with Krebs solution (120 mM NaCl, 2.4 mM KCl, 1.2 mM CaCl_2_, 0.9 mM NaH_2_PO_4_, and 1.4 mM Na_2_HPO_4_, pH 7.4) at a flow of 2 μl/min with a syringe pump. Sample collection started after a 60-min stabilization period. Samples were obtained every 15 min, received in 5.0 μl of perchloric acid solution (0.2 N) and maintained in ice until the end of the experiment. To better compare with the protocol used in behavioral tests, three baseline samples were first obtained, then animals received an amphetamine injection (s.c.), and three samples were collected. By the end of the experiment, samples were kept at −80°C until further analysis. Mice were decapitated under anesthesia, their brains removed, fixed in paraformaldehyde solution (4%), and sliced every 50 μm in a cryostat (Leica apparatus). Slices were stained with cresyl violet for *post hoc* verification of probe location under the light microscope. Only data coming from correct probe placement in the striatum were used for further analysis. EAAT3^*glo*^ *n* = 7 (5M, 2F); EAAT3^*glo*^/CMKII *n* = 9 (5M, 4F).

### Analysis of Striatal Dialysate

Ten microliters of each dialysate sample were injected to the HPLC-ED system with the following equipment: An isocratic pump (model PU-2080 Plus, Jasco Co., Ltd., Tokyo, Japan), a C18 column (model Kromasil 100-3.5-C18, AkzoNobel, Bohus, Sweden), and an electrochemical detector (set at 650 mV, 0.5 nA; model LC-4C, BAS, West Lafayette, IN, United States). The mobile phase, containing 0.1 M NaH_2_PO_4_, 1.0 mM 1-octanesulfonic acid, 1.0 mM EDTA, 1.0% (^*v*^/_*v*_) tetrahydrofuran, and 8.0% (^*v*^/_*v*_) CH_3_CN (pH 3.4) was pumped at a flow rate of 125 ml/min. DA extracellular levels were assessed by comparing the respective peak area and elution time of the sample with a reference standard, and the quantification was performed using a calibration curve for each neurotransmitter (Program ChromPass, Jasco Co., Ltd., Tokyo, Japan). In these conditions, the retention time for dopamine was 9.5 min.

### Identification and Quantification of Dopamine Neurons in the Substantia Nigra *Pars compacta*

Dopamine neurons of the SNc were identified by immunostaining for tyrosine hydroxylase (TH). Mice were anesthetized with a mixture of ketamine/xylazine (50/5 mg/kg, respectively) and cardiacly perfused with 50 ml of PBS followed by 50 ml of 4% paraformaldehyde. The brains were removed and postfixed over night with 4% paraformaldehyde and then changed to a solution of 30% sucrose in PBS. Serial 30-μm-thick slices were obtained in a cryostat (Leica Biosystems, IL, United States) spanning the rostrocaudal range of the SNc and recovered every six slices in the same well of a six multiwell plate. Immunostaining for TH was made with rabbit anti-tyrosine-hydroxylase (657012, Merck Millipore, Darmstadt, Germany), diluted at 1:5,000, and incubated overnight at 4°C in 1% horse serum, 0.03% triton in PBS. Donkey anti-rabbit Cy3 (711165152, Jackson Labs, Bar Harbor, ME, United States) diluted at 1:1,000 was used as secondary antibody and incubated for 1 h in the same solution as primary antibody. After three consecutive rinses with PBS, slices were incubated with DAPI (Thermo Fisher Scientific, Waltham, MA, United States) 300 nM for 5 min and then mounted with Fluromount mounting medium (Sigma-Aldrich, St. Louis, MO, United States). Pictures of SNc sections were obtained using a confocal microscope (Nikon) using a 20 × objective with a resolution of 1,024 × 1,024 pixels, and with z steps of 2 μm. TH cells were counted only if the nucleus was fully covered by the z sections. Z-stacks were collapsed in a single image, and cells were counted using the cell counter plugin of image J and then standardized by the area in mm^2^. EAAT3^*glo*^ *n* = 7 (3M, 4F); EAAT3^*glo*^/CMKII *n* = 11 (8M,3F).

### Quantification of Striatal Dopamine Proteins

Mice were anesthetized with isofluorane in a hermetic saturated chamber, and the brains were quickly removed and rinsed in ice-cold PBS 1 ×. The dorsal striatum was obtained by dissecting a 2-mm-thick slice and immediately stored at −80°C until protein extraction. Proteins were extracted in RIPA lysis buffer with protease inhibitor cocktail. Protein samples (30 μg) were separated by SDS-PAGE on 10% polyacrylamide gels under denaturing conditions (4% stacking gel, 10% resolving gel). Proteins were transferred to nitrocellulose membrane (Cat# 88018, 0.45-μm pore, Thermo Scientific^TM^, Rockford, IL, United States) at 350 mA for 1.5 h. Non-specific sites of membrane binding were blocked with 5% skim milk in T-TBS (0.1% Tween-20, 20 mM TBS, 137 mM NaCl) for 1 h at room temperature. Primary antibodies for immunodetection of dopamine system proteins were selected based on previously published data: 1:1,000 of rabbit anti-D1R (AB1765P, Merck Millipore, Darmstadt, Germany) (Huang et al., 1992; Graham et al., 2015); 1:1,000 of rabbit anti-D2R (AB5084P, Merck Millipore, Darmstadt, Germany) (Galvan et al., 2014; Graham et al., 2015); 1:10,000 of rabbit anti-TH (657012, Merck Millipore, Darmstadt, Germany) (Espinosa et al., 2016); 1:2,000 of rabbit anti-DAT (434-DATEL2, Phosphosolutions, Aurora, CO, United States) (Dib et al., 2018; Alonso et al., 2021). Rabbit anti-GAPDH (G9545, Sigma-Aldrich, St. Louis, MO, United States) at 1:10,000 was used as loading control. As secondary antibody, donkey anti-rabbit conjugated with HRP (711036152, Jackson Immunoresearch Laboratories, Baltimore, MD, United States) diluted 1:5,000 was used. For chemiluminescent detection, we used EZ-ECL kit (SuperSignal^TM^ Femto, Thermo Scientific, Rockford, IL, United States), and the images of the membranes were obtained using a benchtop darkroom (EpiChemi^3^ Darkroom, UVP, Upland, CA, United States). The images were analyzed using Image-J^TM^ software. EAAT3^*glo*^ *n* = 4; EAAT3^*glo*^/CMKII *n* = 4.

### Analysis and Statistics

Locomotor activity and dopamine release were analyzed by two-way repeated measures ANOVA (group × time) followed by Bonferroni posttest. The percentage of grooming events in each phase of grooming chain syntax was analyzed by two-way repeated measures ANOVA (group × phase) followed by Sidak’s multiple comparisons test. Number of grooming events, time spent in grooming behavior, number of dopamine neurons, and levels of dopaminergic proteins were analyzed by unpaired *t*-test. Data are reported as mean ± SEM. Statistical analysis was done using GraphPad Prism v6.0 (GraphPad Software, San Diego, CA, United States), and *p* < 0.05 was considered statistically significant.

## Results

### Increased Spontaneous Grooming Behavior in EAAT3^*glo*^/CMKII Mice Is Accompanied by an Altered Syntax and It Is Insensitive to Amphetamine

**FIGURE 1:**
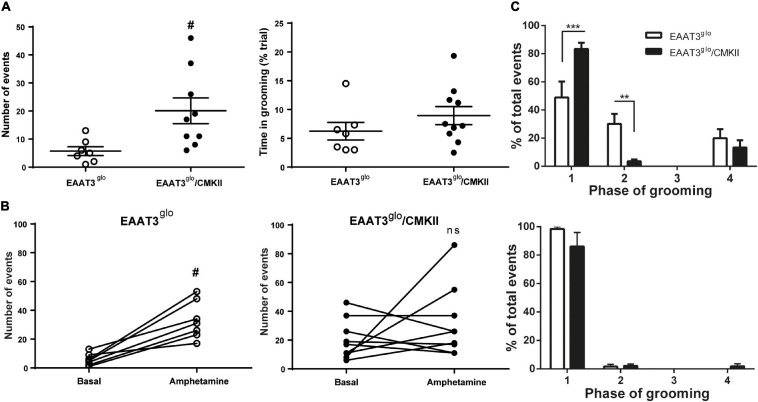
Grooming behavior is increased and its chain syntax altered in EAAT3^*glo*^/CMKII mice. Mice were placed in a cylindrical transparent plexiglass container, and its grooming behavior was video recorded for 10 min in basal conditions and after an acute injection of amphetamine [5 mg/kg subcutaneously (s.c.)]. Analysis of grooming was made by an experimenter blind to the genotype of the mice. **(A)** Comparison of grooming behavior between EAAT3^*glo*^ and EAAT3^*glo*^/CMKII mice in basal conditions. **(B)** Number of grooming events in basal conditions paired with the number of grooming events after acute administration of amphetamine in EAAT3^*glo*^ (left) and EAAT3^*glo*^/CMKII (right) mice. **(C)** Distribution of grooming events in the phases of the grooming-chain syntax in basal (upper panel) and after amphetamine administration (lower panel). EAAT3^*glo*^ *n* = 7 (4M, 3F); EAAT3^*glo*^/CMKII *n* = 9 (3M, 6F). **p < 0.01, ***p > 0.001, according to two-way ANOVA and Sidak’s multiple comparison test. ^#^*p* = 0.0187, according to unpaired *t*-test.

We tested whether EAAT3 overexpression leads to changes in grooming structure evaluating this behavior in trials of 10 min. EAAT3^*glo*^/CMKII mice showed increased number of spontaneous grooming events compared with control littermates (EAAT3^*glo*^ 5.7 ± 1.6 events, *N* = 7; EAAT3^*glo*^/CMKII 20.4 ± 4.6 events, *N* = 9. *P* = 0.0187, according to unpaired *t*-test). However, the time spent in grooming was not different between groups (EAAT3^*glo*^ 6.238 ± 1.529 s, *N* = 7; EAAT3^*glo*^/CMKII 8.950 ± 1.564 s, *N* = 10. *p* = 0.2509, according to unpaired *t*-test) (Figure 1A) suggesting that the duration of individual grooming bouts should be shorter in EAAT3^*glo*^/CMKII mice. We assessed then if EAAT3 overexpression leads to a change in the grooming chain syntax (Figure 1C, upper panel). We found that both EAAT3^*glo*^/CMKII and EAAT3^*glo*^ mice have a higher proportion of events at phases 1 and 2, and a low proportion of events reaching phase 4 [grooming phase: *F*_(__3_, _60__)_ = 58.38, *p* < 0.0001; genotype: *F*_(__1_, _60__)_ = 0.006677, *p* = 0.9351; interaction: *F*_(__3_, _60__)_ = 11.57, *p* < 0.0001; two-way ANOVA]. Interestingly, EAAT3^*glo*^/CMKII mice have a significantly higher proportion of events in phase 1 and a lower proportion in phase 2 compared with controls (phase 1: *p* < 0.0001; phase 2: *p* < 0.01. Sidak’s multiple comparisons test), suggesting that EAAT3-overexpressing mice get stuck on the initiation phase of grooming without completing the full behavior. To assess whether amphetamine has a differential effect on grooming due to increased EAAT3 expression, we acutely administered amphetamine (5.0 mg/kg) to EAAT3^*glo*^/CMKII and control mice. Interestingly, amphetamine significantly increased the number of grooming events only in controls (EAAT3^*glo*^: basal events 5.71 ± 1.57, amphetamine events 33.14 ± 4.96; *p* = 0.0156, paired *t*-test), while no significant effect was observed in EAAT3-overexpressing mice (EAAT3^*glo*^/CMKII: basal events 20.11 ± 4.58, amphetamine events 31.89 ± 8.20; *p* = 0.5781, paired *t*-test) (Figure 1B). Moreover, the syntax chain of grooming phases was shifted in control EAAT3^*glo*^ mice toward almost all events classified in phase 1, resembling what happens with EAAT3^*glo*^/CMKII mice in basal conditions. On the other hand, EAAT3^*glo*^/CMKII was maintained in phase 1 [grooming phase: *F*_(__3_, _60__)_ = 215.0; *p* < 0.0001; genotype: *F*_(__1_. _60__)_ = 0.6450; *p* = 0.4251; interaction: *F*_(__3_, _60__)_ = 1.125; *p* = 0.3462; two-way ANOVA], while multiple comparisons test did not show any difference between genotypes (Figure 1B, bottom panel). Together, the data suggest that EAAT3 overexpression converts grooming from a spontaneous behavior to an “amphetamine-like” behavior.

### Amphetamine-Induced Increase in Locomotor Activity Is Not Modified by EAAT3 Overexpression

**FIGURE 2:**
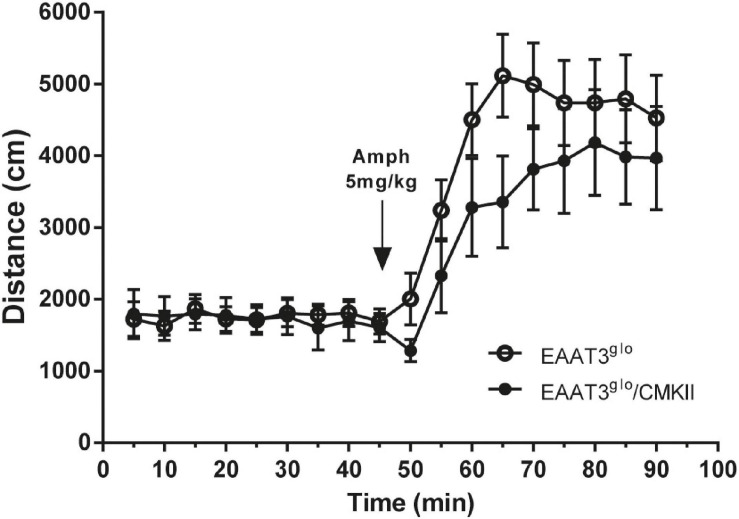
Basal and amphetamine-induced locomotion is unaltered in EAAT3^*glo*^/CMKII mice. Mice were placed in an open-field arena (40 × 40 × 35 cm) and their horizontal trajectory was video recorded in basal conditions and after acute amphetamine (5 mg/kg s.c.). Graph shows the cumulative distance traveled by mice in 5 min blocks during 45 min before and after amphetamine. EAAT3^*glo*^ *n* = 11 (6M, 5F); EAAT3^*glo*^/CMKII *n* = 8 (5M, 3F).

We wondered whether increased EAAT3 expression influences the amphetamine-induced locomotor behavior. To assess this, we measured locomotor activity in an open-field arena in basal conditions and after amphetamine administration. We also analyzed the first 5 min in the arena to evaluate anxiety. As we previously reported (Delgado-Acevedo et al., 2019), EAAT3^*glo*^/CMKII mice spent significantly less time in the open area of the arena (EAAT3^*glo*^: 52.55 ± 11.33 s, *N* = 11; EAAT3^*glo*^/CMKII: 19.38 ± 4.89 s, *N* = 8; *p* = 0.0300, unpaired *t*-test), indicating increased anxiety-like behavior (Supplementary Figure 1). As shown in Figure 2, spontaneous locomotor activity was not modified by EAAT3 overexpression as basal locomotor activity was similar between genotypes. Amphetamine administration significantly increased locomotor activity to the same extent in both genotypes, although maximal locomotor effect was delayed by 15 min in EAAT3^*glo*^/CMKII mice in comparison with EAAT3^*glo*^ [time: *F*_(__17_, _306__)_ = 16.14; *p* < 0.0001; genotype: *F*_(__1_, _306__)_ = 10.81; *p* = 0.0011; interaction: *F*_(__17_, _306__)_ = 0.7600. *p* = 0.7387; two-way ANOVA]. Together, the data indicate that the increased basal grooming observed in EAAT3^*glo*^/CMKII mice is not associated with modifications in horizontal locomotor activity and that although amphetamine does not affect grooming, the induction of locomotion is preserved in this mouse model.

### Decreased Basal Dopamine Release and Increased Amphetamine-Induced Dopamine Release in EAAT3-Overexpressing Mice

**FIGURE 3:**
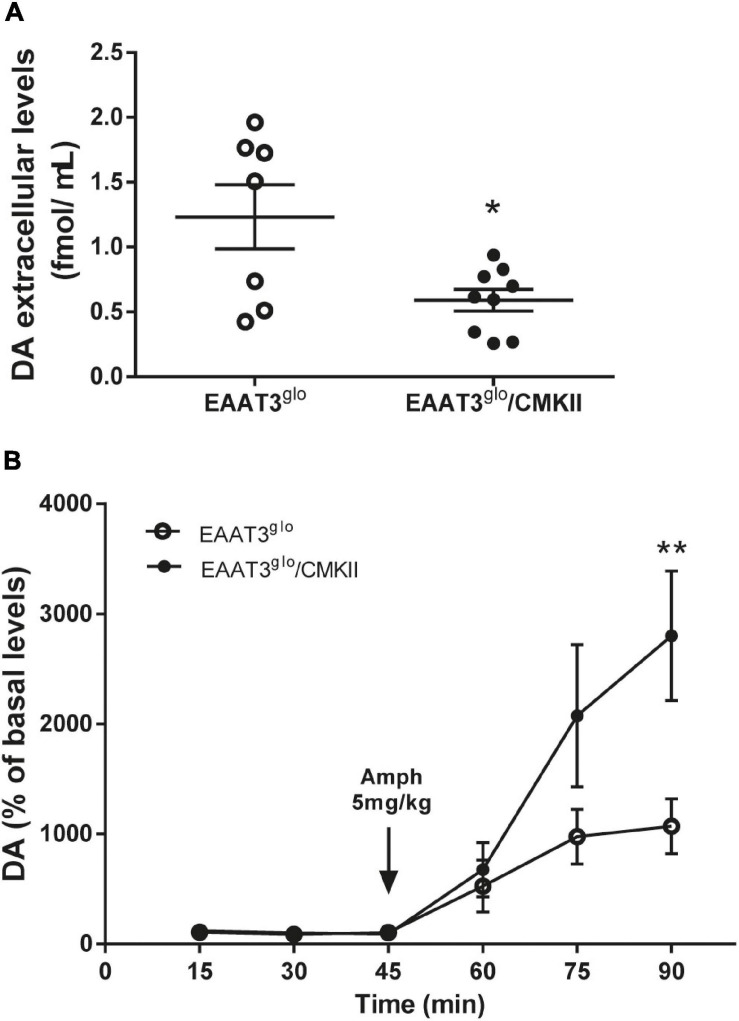
Decreased basal dopamine (DA), but enhanced amphetamine-induced DA levels in EAAT3^*glo*^/CMKII mice. *In vivo* microdialysis was done in the striatum of anesthetized mice. Samples were collected every 15 min; three samples were taken in basal conditions and after acute administration of amphetamine (5 mg/kg s.c.). **(A)** Basal DA extracellular concentration in the striatum. Graph shows the average of the three samples taken for basal DA concentration. *p = 0.0163 according to unpaired *t*-test. **(B)** Temporal course of amphetamine-induced DA release. Data are expressed as percentage of basal DA levels. **p < 0.01 according to two-way ANOVA and Bonferroni’s posttest. EAAT3^*glo*^ *n* = 7 (5M, 2F); EAAT3^*glo*^/CMKII *n* = 9 (5M, 4F).

To evaluate whether behavioral effects of EAAT3 overexpression are correlated with modifications on dopaminergic transmission, we performed *in vivo* brain microdialysis experiments in the striatum in basal conditions and after an amphetamine challenge. We carried out microdialysis experiments in isofluorane-anesthetized mice. We used isofluorane because it induces a stable level of anesthesia during an extended period. As urethane, it does not modify dopamine dynamics (Sabeti et al., 2003; Brodnik and España, 2015). We did not observe any difference in sensitivity to induce anesthesia between genotypes, as every mouse needed around 3% isofluorane in 0.5 L/min of airflow for induction and between 1 and 2% isofluorane in 0.5 L/min airflow for maintenance. We found that basal dopamine release was reduced in EAAT3^*glo*^/CMKII mice compared with controls (EAAT3^*glo*^: 1.233 ± 0.247 fmol/μl, *N* = 7; EAAT3^*glo*^/CMKII: 0.5912 ± 0.08, *N* = 9; *p* = 0.0163, unpaired *t*-test) (Figure 3A). This indicates that the increased grooming behavior in these mice and its alteration of the chain syntax are accompanied by reductions in tonic DA release. Acute amphetamine administration increased dopamine extracellular levels in both groups. Surprisingly, this increase was significantly higher in EAAT3^*glo*^/CMKII mice in comparison with their control littermates [time: *F*_(__5_, _84__)_ = 12.99, *p* < 0.0001; genotype: *F*_(__1_, _84__)_ = 7.410, *p* = 0.0079; interaction: *F*_(__5_, _84__)_ = 2.703, *p* = 0.0258; two-way ANOVA (Figure 3B)]. *Post hoc* test revealed that the enhanced DA release in EAAT3^*glo*^/CMKII mice reached significance 45 min after amphetamine administration (min 90, *p* < 0.001, Bonferroni’s posttest). Overall, the data indicate that the lack of effect of amphetamine on grooming behavior of EAAT3^*glo*^/CMKII mice is not associated with a decreased ability of amphetamine to release DA, but rather to unexpectedly sensitize DA release, suggesting that cellular mechanisms arise to set the grooming behavior of EAAT3^*glo*^/CMKII at a maximum point so that this behavior cannot be higher even though DA release increases.

### EAAT3-Overexpressing Mice Have Increased Expression of DA Type 2 Receptors in the Striatum

**FIGURE 4:**
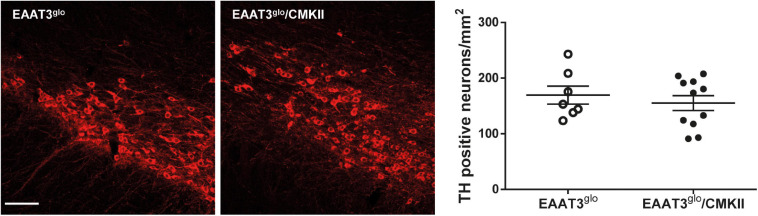
The number of DA neurons is preserved in EAAT3^*glo*^/CMKII mice. Immunohistochemistry was made against tyrosine hydroxylase (TH) in 30-μm-thick coronal sections spanning the rostrocaudal axis containing the substantia nigra pars compacta (SNc); the number of TH-positive neurons was quantified and standardized by area in mm^2^. Images depict 10 × representative maximum intensity projections of flattened z-stack pictures. Scale bar: 50 μm. EAAT3^*glo*^ *n* = 7 (3M, 4F); EAAT3^*glo*^/CMKII *n* = 11 (8M, 3F).

**FIGURE 5:**
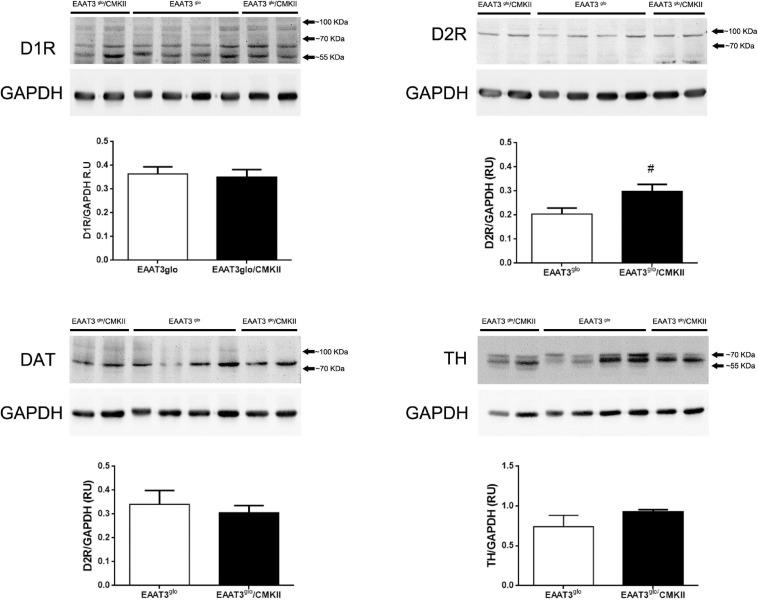
Increased expression of DA D2R in the striatum of EAAT3^*glo*^/CMKII mice. Thirty micrograms of striatal protein extracts was processed for the immunodetection and quantification of proteins of the dopaminergic system. Western blot images show the individual bands of protein samples obtained from four mice per group; protein band quantification is depicted below. #p = 0.0286, according to unpaired *t*-test. EAAT3^*glo*^ *n* = 4; EAAT3^*glo*^/CMKII *n* = 4.

It has been reported that EAAT3 KO mice have fewer DA neurons at 12 months old (Berman et al., 2011) but not at 3 months old (Berman et al., 2011; Zike et al., 2017). Thus, it is possible that EAAT3 overexpression impacts on the number of these midbrain neurons. To address this question, we performed immunohistochemistry and counted the number of TH-positive neurons in serial sections of the SNc. As shown in Figure 4, EAAT3^*glo*^/CMKII and control mice have the same number of DA neurons per area in the SNc (EAAT3^*glo*^: 160.5 ± 16.2, *N* = 7; EAAT3^*glo*^/CMKII: 155.0 ± 13.4, *N* = 11; *p* = 0.5134), indicating that increased DA release by amphetamine is not due to the presence of more DA neurons. We then assessed the expression DA system proteins in the striatum, the target area of DA neurons from SNc. As shown in Figure 5, similar levels of expression were found for D1R, DAT, and TH between EAAT3^*glo*^/CMKII and control mice. Interestingly, we found that the expression of D2R was significantly higher in EAAT3^*glo*^/CMKII mice (EAAT3^*glo*^: 0.2050 ± 0.0248, *N* = 4; EAAT3^*glo*^/CMKII: 0.2988 ± 0.0288, *N* = 4; *p* = 0.0490), suggesting that EAAT3 overexpression is related to an imbalance between D1R direct and D2R indirect- striatal pathways.

## Discussion

In this work, we characterized the behavioral, neurochemical, and molecular components of the dopamine system in basal conditions and after acute administration of amphetamine in EAAT3glo/CMKII, a recently developed mouse model that overexpresses the neuronal glutamate transporter EAAT3 in principal forebrain neurons. In addition to replicate previous data showing increased grooming behavior (Delgado-Acevedo et al., 2019), we found that acute amphetamine administration does not further stimulate this behavior. Interestingly, most of the spontaneous grooming events of EAAT3^*glo*^/CMKII mice remain in phase 1, without progressing to the next stages of the chain syntax (Cromwell et al., 1998; Berridge et al., 2005; Kalueff et al., 2007). In control mice, amphetamine administration induced an increase in grooming events and a shift toward phase 1 of the grooming chain syntax. Together, the data suggest that EAAT3 overexpression alters grooming rendering it similar to an amphetamine-like behavior. Despite the increased spontaneous grooming behavior, basal dopamine levels were decreased in EAAT3^*glo*^/CMKII mice. Surprisingly, amphetamine administration induced a higher release of dopamine in this model. At the molecular level, EAAT3 overexpression induced an increase of the expression of dopamine D2R in the striatum. Overall, the data suggest that the increased EAAT3 expression impacts on dopamine system indicating that the increased and altered syntax of grooming is accompanied by a decreased tonic release of dopamine in the striatum and enhanced expression of dopamine receptors in the indirect striatal pathway. On the other hand, the increased EAAT3 expression sensitizes the response of DA neurons to amphetamine.

We previously reported that EAAT3^*glo*^/CMKII mice display increased repetitive (including grooming) and anxiety-like behaviors as well as altered corticostriatal synaptic function (Delgado-Acevedo et al., 2019). In the present work, we found that the increased spontaneous grooming behavior is due to increased number of grooming bouts that mostly remain in phase 1 of the grooming-chain syntax (Kalueff et al., 2007), without completing the entire sequence (Figures 1A,C). This suggest that the increased EAAT3 expression impacts mechanisms that facilitate the initiation of grooming, while at same time, it shunts the progression to the following phases of this behavior. The execution of a complete grooming behavior relies on the integrity of dopamine nigrostriatal neurotransmission (Berridge, 1989; Berridge et al., 2005). Indeed, depletion of dopamine in the nigrostriatal pathway by injection of 6-OHDA as well as ablation of dopamine D1 receptor (D1R) disrupt grooming behavior (Berridge, 1989; Cromwell et al., 1998; Pelosi et al., 2015). EAAT3 is expressed in dopamine neurons (Plaitakis and Shashidharan, 2000; Sidiropoulou et al., 2001; Berman et al., 2011; Underhill et al., 2014) where its ablation causes modifications on this system including a decrease in the number of dopamine neurons in the SNc (Berman et al., 2011), decreased basal dopamine levels (Zike et al., 2017), and expression of D1R in the striatum (Bellini et al., 2018). Thus, we hypothesized that EAAT3 overexpression might modify basal dopamine transmission to underlie increased grooming behavior. Microdialysis experiments in the striatum show decreased basal dopamine levels and increased expression of D2R in EAAT3^*glo*^/CMKII mice compared with controls (Figures 3A, 5). Low basal DA levels have been also reported in other preclinical models of compulsive-checking behavior induced by repeated administration of the D2R agonist, quinpirole (Koeltzow et al., 2003; De Haas et al., 2011; Escobar et al., 2015, 2017). A decreased level of basal dopamine is not explained by less dopamine neurons as the number of TH-positive neurons was not modified by EAAT3 overexpression (Figure 4). Our results suggest that by increasing EAAT3 expression in midbrain dopamine neurons, local somatic levels of glutamate are reduced, therefore, reducing the firing of DA neurons. Interestingly, striatal dopamine depletion induced by 6-OHDA injection did not reduce the number of grooming bouts, but its progression to following phases of the grooming-chain syntax (Berridge, 1989). Thus, it is possible that reduced levels of striatal DA contribute to the alterations in grooming behavior found in EAAT3^*glo*^/CMKII mice.

Based on our previous findings of increased grooming in EAAT3^*glo*^/CMKII mice, our present work focused on the characterization of dopamine neurons in the SNc, where no changes were found in the number of TH-positive neurons. However, we did not evaluate alterations in VTA. In this regard, the reduced number of dopamine neurons in the SNc of EAAT3 KO mice, reported by Berman and colleagues, was only evident in aged (1 year old), but not in younger (3- to 6-month old) mice (Berman et al., 2011), and it was restored by the administration of N-acetylcysteine, indicating that the effect was due to the lack of cysteine uptake via EAAT3 and an enhancement of the cumulative oxidative damage over time. Using animals at a similar age than in our study, Zike and colleagues found no changes in the number of dopamine neurons of SNc or VTA (Zike et al., 2017). Future experiments should address the impact of EAAT3 overexpression on the number of dopamine neurons in the VTA and mesocorticolimbic function in EAAT3^*glo*^/CMKII mice.

We found increased expression of D2R in the striatum of EAAT3^*glo*^/CMKII mice (Figure 5). D2R has two splicing variants, the short (D2RS) isoform is present presynaptically at dopamine terminals, while the long (D2RL) is present postsynaptically at medium spiny neurons of the indirect pathway (Sesack et al., 1994; Usiello et al., 2000). In this work, we quantified the expression of total D2R; therefore, we could not unravel if the increased EAAT3 expression has a differential impact on D2R isoforms. When activated, D2RS decreases dopamine levels by increasing dopamine reuptake and by decreasing the synthesis and firing of dopamine neurons (Carlson et al., 1986; Napier et al., 1986; Lacey et al., 1987; O’Hara et al., 1996). Thus, an enhanced D2RS expression could explain the reduced basal DA level observed in our mouse model. Whether an increase in D2R triggers a reduction in basal DA, or if the reduced DA triggers a homeostatic increase in D2R remains to be investigated. On the other hand, increased expression of D2RL isoform should be reflected on behavioral traits. Upon activation, D2RL inhibits the activity MSNs of indirect pathway leading to movement facilitation. Therefore, increasing the expression of this receptor could magnify or induce repetitive movements. Indeed, repeated activation of D2R induces sensitization of locomotor activity and compulsive-like behaviors in rats and mice (Szechtman et al., 1998, 2017; Escobar et al., 2015; Asaoka et al., 2019; Sun et al., 2019), which is paralleled with an increased expression of D2R in the high-affinity state (Perreault et al., 2007). Moreover, Deer and C58 mice lines, which naturally develop repetitive-like movements, have a decreased neuronal activity of the indirect pathway (Tanimura et al., 2010), which is restored upon concomitant administration of a D2R antagonist, an adenosine 2A receptor agonist and a mGluR5 receptor-positive allosteric modulator that are able to alleviate the increased repetitive behavior (Muehlmann et al., 2020). To the best of our knowledge, there are no studies addressing grooming syntax in Deer or C58 mice.

Other neurotransmitter systems could also be impacted by EAAT3 overexpression and contribute to the phenotype of EAAT3^*glo*^/CMKII mice. In particular, EAAT3 is known to be present in norepinephrine neurons, where amphetamine also stimulates its endocytosis (Underhill et al., 2020). Future studies should investigate potential alterations in norepinephrine as well as serotonin neurotransmission in EAAT3^*glo*^/CMKII mice, based on the effectiveness of the current pharmacotherapy targeting these neurotransmitter systems in humans.

Together, the data indicate that chronic inhibition of the indirect striatal pathway triggers the development of compulsive-like behavior and suggest that the increased expression of D2R could mediate the development of the altered grooming behavior observed in the EAAT3^*glo*^/CMKII mice. Additionally, increased grooming behavior can be ascribed to the enhanced contribution of GluN2B containing NMDA receptors (Delgado-Acevedo et al., 2019). Indeed, antagonism of NMDA receptors reduced grooming and other repetitive behaviors in rodents prenatally exposed to valproic acid, an animal model of autism (Kang and Kim, 2015; Kim et al., 2017). Therefore, it is plausible to hypothesize that the alteration in GluN2B–NMDA receptor, together with reduced basal DA and enhanced D2R expression, impacts on an augmented and structurally different grooming behavior.

It is known that EAAT3 mediates the effect of the psychostimulant amphetamine on the dopamine system. Acute amphetamine induces a decrease in the surface expression of EAAT3 in midbrain dopamine neurons, which enhance glutamate receptors currents (Underhill et al., 2014; Li et al., 2017). Moreover, amphetamine administration to EAAT3 KO mice induces a lower increase in locomotion and grooming behavior and a lower increase in dopamine extracellular levels compared with controls (Zike et al., 2017). We found that, in EAAT3^*glo*^/CMKII mice, amphetamine induced an increase in locomotor activity that was similar to control mice; however, amphetamine increased grooming only in control mice, enhancing the total number of events and their distribution to phase 1 of the grooming chain syntax. EAAT3^*glo*^/CMKII mice challenged with amphetamine remained on the same number and phase of grooming events, indicating that increased EAAT3 expression triggers a “ceiling” effect on grooming. Contrary to its behavioral effect, amphetamine induced a significantly higher increase in extracellular dopamine levels in EAAT3^*glo*^/CMKII mice compared with controls, indicating that EAAT3 overexpression renders dopamine neurons more responsive to amphetamine, triggering a higher dopamine releasability in the striatum. This result is surprising given the known parallel between higher dopamine extracellular levels with increased locomotion and stereotypy (Pierce and Kalivas, 1997; Kwiatkowski et al., 2019), suggesting that increased EAAT3 overexpression induces glutamatergic mechanisms that affect behavior in response to higher dopamine release. The exposure of dopamine neurons to amphetamine induces the endocytosis of DAT and EAAT3, a phenomenon dependent of the activation of the small GTPase *RhoA* and protein kinase A (PKA) (Underhill et al., 2014). The underlying mechanism includes the entry of amphetamine thorough DAT (Underhill et al., 2014) and the binding to the trace amine-associated receptor, TAAR1, an intracellular G protein-coupled receptor that activates these signaling pathways (Underhill et al., 2021). The decrease in the surface expression of DAT should contribute to the effects of amphetamine to enhance local dopamine extracellular levels. In the same direction, the amphetamine-driven decrease in surface EAAT3 increases local extracellular glutamate and augments the amplitude of NMDA eEPSCs (Underhill et al., 2014), particularly GluN2B-containing NMDA receptors (Li et al., 2017). It is possible that in EAAT3^*glo*^/CMKII mice, the challenge with amphetamine induces the endocytosis of more EAAT3 units compared with controls; this could be sensed by cells as a higher increase in glutamate extracellular levels, which would influence on the level of activation of NMDA receptors. Importantly, activation of NMDA receptors induces and controls the duration and frequency of burst firing in dopamine neurons (Johnson et al., 1992; Chergui et al., 1993; Pearlstein et al., 2015). Burst firing determines phasic and transient increases in dopamine release in target areas that determines salient stimuli. Recently, it has been shown that a single burst of electrical stimulation of midbrain dopamine neurons induces a long-lasting dopamine release in the striatum, which is measurable by microdialysis experiments (Lohani et al., 2018). Together, the data suggest that the enhanced release of dopamine in the striatum seen in EAAT3^*glo*^/CMKII mice can also be explained by modifications of glutamate transmission that would lead to a facilitation of phasic activity of dopamine neurons, indicating that the levels of EAAT3 in midbrain dopamine neurons could impact on their firing mode and, by doing so, influence the dopamine transmission in target areas and ultimately behavior.

## Conclusion

In conclusion, in this study, we found that EAAT3 overexpression impacts on dopamine transmission with pre- and postsynaptic effects in the striatum. These modifications could underlie some of the compulsive behaviors observed in EAAT3^*glo*^/CMKII mice and points to EAAT3 as a key mediator in the interplay between dopamine and glutamate transmission.

## Data Availability Statement

The raw data supporting the conclusions of this article will be made available by the authors, without undue reservation.

## Ethics Statement

The animal study was reviewed and approved by Bioethics Committee of the Universidad de Valparaiso Protocol No. BEA138-19.

## Author Contributions

AE and PRM conceived and designed the study. AE, JM-P, and FS-O performed the experiments, collected, and analyzed the data. AE wrote the first manuscript. PRM and RS-Z wrote sections of the manuscript. All authors participated in the manuscript revision as well as read and approved the submitted version.

## Conflict of Interest

The authors declare that the research was conducted in the absence of any commercial or financial relationships that could be construed as a potential conflict of interest.

## Supplementary Material

Supplementary Figure 1 EAAT3^*g**lo*^/CMKII mice show anxiety-like behavior. Mice were placed in an open-field arena (40 × 40 × 35 cm) and allowed to freely explore for 5 min. The time spend in center (20 × 20 cm) was analyzed. *P* = 0.03, according to unpaired *T*-test. EAAT3^*g**lo*^ *n* = 11 (6M, 5F); EAAT3^*g**lo*^/CMKII *n* = 8 (5M, 3F).
