# Supplementary figures and images for: Altered Grooming Syntax and Amphetamine-Induced Dopamine Release in EAAT3 Overexpressing Mice
Source: Front Cell Neurosci. 2021 Jun 21;15:661478. doi: 10.3389/fncel.2021.661478 (PMC8255620; doi:10.3389/fncel.2021.661478)

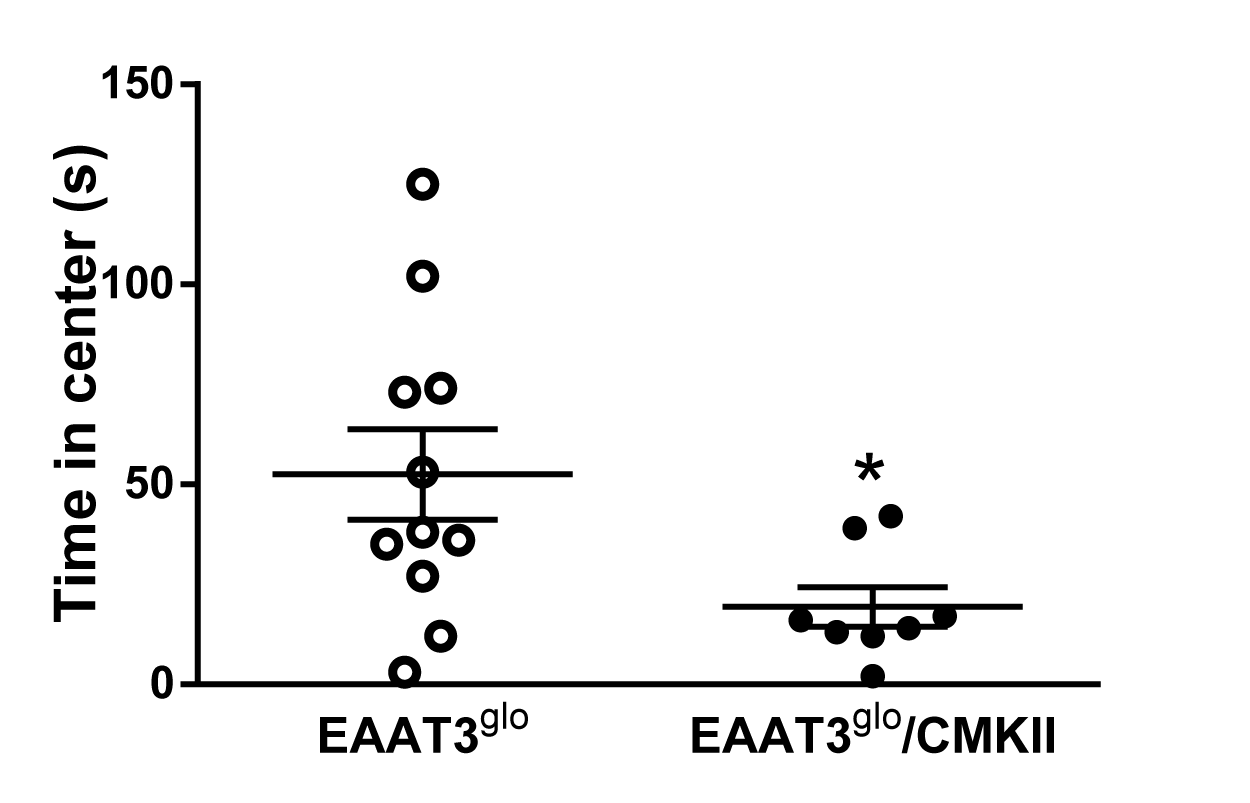

Supplement: Supplementary Figure 1 — EAAT3glo/CMKII mice show anxiety-like behavior. Mice were placed in an open-field arena (40 × 40 × 35 cm) and allowed to freely explore for 5 min. The time spend in center (20 × 20 cm) was analyzed. P = 0.03, according to unpaired T-test. EAAT3glo n = 11 (6M, 5F); EAAT3glo/CMKII n = 8 (5M, 3F). [file Image_1.TIF]
